# Underwater endoscopic mucosal resection of serrated adenomas

**DOI:** 10.6061/clinics/2018/e339

**Published:** 2018-09-24

**Authors:** Dalton Marques Chaves, Hélcio Pedrosa Brito, Lumi Tomishige Chaves, Rodrigo Azevedo Rodrigues, Beatriz Mônica Sugai

**Affiliations:** I Endoscopia, Fleury Medicina e Saude, Sao Paulo, SP, BR; II Gastroenterologia, Hospital das Clinicas HCFMUSP, Faculdade de Medicina, Universidade de Sao Paulo, Sao Paulo, SP, BR; III Faculdade de Medicina, Universidade Federal de Sao Paulo, Sao Paulo, SP, BR

**Keywords:** Adenoma, Colorectal Neoplasm, Endoscopic Mucosal Resection, Polyps, Colon

## Abstract

**OBJECTIVES:**

Serrated polyps, which are considered to be precursors of colorectal carcinoma, include hyperplastic polyps, sessile serrated adenomas and traditional serrated adenomas. With the exception of hyperplastic polyps, all of these lesions must be removed. This study sought to examine whether underwater endoscopic mucosal resection is a safe and effective technique for treating serrated polyps.

**METHODS:**

Cases in which patients were submitted for underwater endoscopic mucosal resection and histologically diagnosed with sessile serrated adenoma were prospectively registered.

**RESULTS:**

The median patient age was 54.5 years (range, 48 to 72 years), and the patients included 4 men (28.5%) and 10 women (71.5%). One lesion (6.2%), 10 lesions (62.5%), 1 lesion (6.2%), 3 lesions (18.8%) and 1 lesion (6.2%) were found in the cecum, the ascending colon, the hepatic flexure, the transverse colon and the descending colon, respectively. The median lesion size was 20 mm (range, 10 to 35 mm). Eight lesions (50%) were removed *en bloc*, and the remaining eight lesions (50%) were removed using a piecemeal technique. None of the cases were complicated by perforation or delayed bleeding.

**CONCLUSION:**

Underwater resection could be a feasible, safe and effective alternative for the resection of sessile serrated adenomas.

## INTRODUCTION

Serrated polyps (SPs) are considered to be precursors of colorectal carcinoma (CRC) and represent a group of polyp subtypes with different colonoscopic, histologic and molecular characteristics; in particular, SPs have been divided into hyperplastic polyps (HPs), sessile serrated adenomas (SSAs) and traditional serrated adenomas (TSAs) 1,2. During colonoscopy, these SP subtypes have characteristic appearances: HPs are typically small and located in the distal colon; SSAs are typically located in the proximal colon, are flat, and have a mucus cap adherent to their surface; and TSAs are found mainly in the left colon, are bulkier than other SPs, and are easy to identify due to their protuberant pinecone shape and tendency to be pedunculated or sessile 1,2. With the exception of HPs, all of these lesions must be removed 1-3.

In 2012, Binmoeller 4 suggested lesion resection that involved filling the colon lumen with water instead of utilizing submucosal injections. As an additional potential advantage of this approach, the refractive index of water contributes to objects appearing larger underwater 5 and should therefore increase the chances of identifying the boundaries and characteristics of lesions.

Given these observations, the aims of this study were to determine whether underwater resection is feasible, safe and effective for removing SPs.

## PATIENTS AND METHODS

This study included consecutive patients referred to our service for screening colonoscopy who underwent underwater endoscopic resection (UEMR) for treatment of an SSA of ≥10 mm. From June 2014 to February 2017, 14 patients underwent UEMR and were prospectively registered in our database. Inclusion criteria were the performance of UEMR by a single endoscopist (D.M.C.), a diagnosis of flat lesions and a histological diagnosis of SSA.

Colonoscopy was conducted using a colonoscope with an accessory channel (CF-H180AI; Olympus®, Tokyo, Japan) and an auxiliary water jet (CF-H180AL; Olympus Medical Systems®, Center Valley, PA) to immerse the lesion underwater. The procedure was performed with the patient in the left lateral position under monitored conscious sedation anesthesia. Scopolamine butylbromide was intravenously administered if necessary to decrease peristalsis. Lesions were measured using the diameter of an open snare.

**Figure 1:**
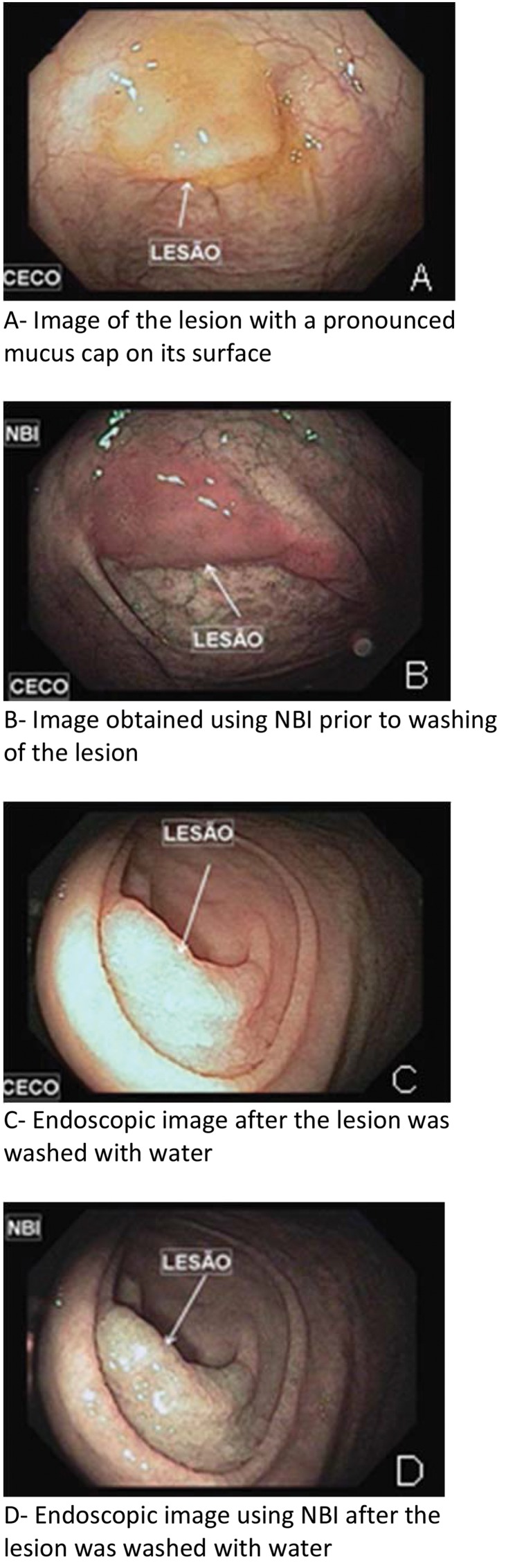
Serrated adenoma in the cecum.

**Figure 2:**
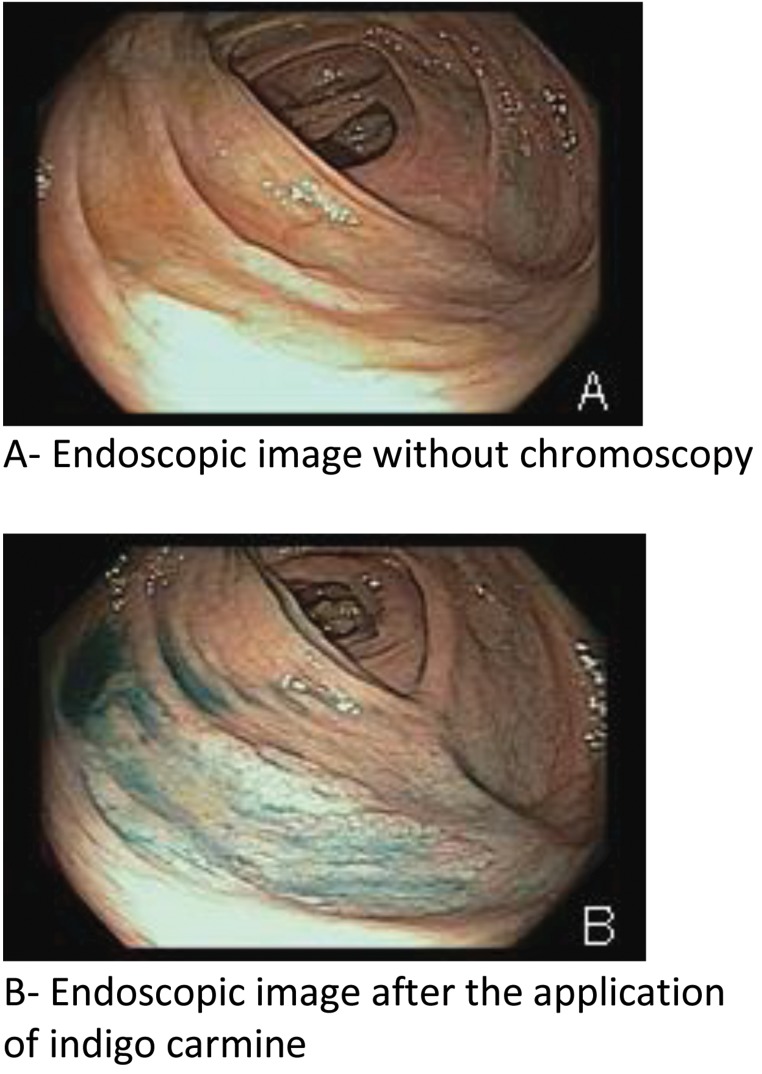
Serrated adenoma in the ascending colon.

After a lesion was identified in the right colon (Figures 1 and 2), air was evacuated, and water was infused to immerse the lesion. The margins of the lesion were identified using high-definition narrow-band imaging (NBI). No diathermic demarcation was performed prior to resection. Resection was conducted by catching the lesion with a snare under direct view. The margin of resection was reviewed using the NBI system. If necessary, piecemeal resection was performed. Lesion remnants in margins that were too small to snare were removed via hot biopsy.

Resections were performed using a multifilament snare (15-25 mm) in Endocut mode (ERBE VIO 200®) with the following settings: effect 3, interval 6, duration 1, 45 W.

## RESULTS

Lesion characteristics and resection details are displayed in Table 1. Two patients had two lesions each. The median patient age was 54.5 years (range, 48 to 72 years), and the patients included 4 men (28.5%) and 10 women (71.5%). One lesion (6.2%), 10 lesions (62.5%), 1 lesion (6.2%), 3 lesions (18.8%), and 1 lesion (6.2%) were found in the cecum, the ascending colon, the hepatic flexure, the transverse colon and the descending colon, respectively. The median lesion size was 20 mm (range, 10 to 35 mm). Eight lesions (50%) were removed *en bloc*, whereas eight lesions (50%) were removed using a piecemeal technique. None of the cases were complicated by perforation or delayed bleeding.

## DISCUSSION

The incidence of serrated lesions in the population is 5% to 8% (compared with 30% to 40% for adenomas) 6,7; moreover, with the exception of HPs, SPs are considered precursors of CRC due to BRAF mutation and CpG island methylator phenotype (CIMP) with or without microsatellite instability (MSI). Serrated sessile adenomas account for 15% to 20% of SPs and 9% of all types of polyps 3. Recommendations suggest that with the exception of diminutive HPs, serrated lesions must be removed during colonoscopy 1-3. Standard endoscopic resection techniques for these lesions include mucosectomy using electrocautery with prior submucosal injection for large lesions and cold snare techniques without submucosal injection for small lesions (<10 mm) 8-10. Although the use of endoscopic mucosal resection (EMR) techniques is successful for resecting large lesions, local residual and recurrent neoplasia can occur 11.

A previous study showed that endoscopic piecemeal resection (EPR) for sessile polyps was associated with residual polyps in up to 55% of cases 8, and a study involving long-term follow-up of sessile adenomas larger than 2 cm indicated that related macroscopic residual adenomas were found in 17.6% of cases 12.

Binmoeller 4 observed that water immersion made the mucosa and submucosa float away from the *muscularis propria* on endoscopic ultrasound and suggested that water immersion for the resection of larger lesions would allow for the removal of colorectal polyps without submucosal injection. In his series, which included a total of 62 lesions with a median size of 30 mm, an extremely low recurrence rate was observed. When water immersion was used to treat 24 patients with adenomas in the appendiceal orifice 13, 4 patients (20%) were referred to surgery, including 3 patients with UEMR failure due to an inability to exclude adenomatous extension into the appendix during the index procedure and 1 patient with invasive adenocarcinoma in the UEMR specimen; however, only 2 of the 20 patients had residual adenoma.

Because the refractive index of water is 4/3 that of air 5, immersing lesions in water increases the probability of identifying additional characteristics and accurately defining margins; moreover, this approach is associated with an extremely low incidence of perforation due to mucosectomy. In contrast, after saline submucosal injection, lesions sometimes become more difficult to snare, and their borders may be more challenging to identify.

Chromoendoscopy and the use of high-definition endoscopes are imaging enhancement methods that should be considered when lesions may be premalignant. Pohl J. 14 found that chromoendoscopy increased the detection rate for adenomas, flat adenomas and serrated lesions. In a retrospective study, Hazewinkel et al. 15 observed that for white-light endoscopy (WLE), indistinctive borders and cloud-like surfaces are 2 characteristics that independently predict SSAs. With NBI, an irregular shape and dark spots within crypts are also signs of SSAs; these traits exhibit a sensitivity, a specificity and an overall accuracy of 75%, 79% and 77%, respectively, for NBI and 89%, 96% and 93%, respectively, for WLE. Our experience using underwater NBI showed that water immersion resulted in better demarcation of SSAs and contributed to a low incidence of residual lesions. One limitation of our study was a lack of long-term endoscopic follow-up. In addition, although this finding was not directly related to the objective of our study, none of 5 patients that returned for follow-up colonoscopy (at 3 months to 3 years after resection) had residual lesions.

Moussata D et al. 3 reported that most patients with SSAs are females with lesions larger than 5 mm located in the proximal colon (73%), whereas Lieberman et al. 16 reported that 80% of SSAs were in the proximal colon. In the latter study, without specifications regarding the resection technique used, patients with baseline polyps of 10–19 mm had an increased risk of advanced neoplasia compared with that of patients with 5 mm adenomas (15.9% *vs* 7.7%; OR, 2.27; 95% CI, 1.84–2.78). This finding suggests that if there is any question regarding a polyp's complete removal (i.e., if piecemeal resection is required), early follow-up colonoscopy is warranted.

Complications such as perforation and bleeding can occur after the resection of any lesion, although reports 4,13,17,18 have described UEMR as an easy and safe procedure. In 2016, Ponugoti 19 reported the first case of UEMR-associated perforation; this case involved a lesion of >30 mm in the proximal ascending colon. The resection was performed in retroflexion, and the author believes that this aspect of the resection may have been the reason for perforation.

Optimal resection techniques have not yet been defined for SSAs for reasons that include these lesions' morphologies and indistinct margins; however, underwater resection could be a safe and effective alternative to currently utilized techniques.

## AUTHOR CONTRIBUTIONS

Chaves DM was responsible for the oversight of the group, formulation of aims, design of methods, performance of endoscopic procedures, sequential and final review of the manuscript. Brito HP, Chaves LT and Rodrigues RA searched for and reviewed the articles, were responsible for the manuscript writing, preparation and creation of the published work. Sugai BM searched for and reviewed the articles and was responsible for the manuscript writing, provision of materials and patients.

## Figures and Tables

**Table 1:** Patient and treatment characteristics.

Patient #	Sex	Age (years)	Location	Size (mm)	Fragments	R0 resection	Perforation or delayed bleeding	Histology
1	Female	48	Descending colon	10	1	Yes	No	Sessile serrated adenoma
2	Female	66	Ascending colon	15	2	Not available	No	Sessile serrated adenoma
3	Female	50	Transverse colon	25	4	Not available	No	Sessile serrated adenoma
4	Male	51	Ascending colon	20	2	Not available	No	Sessile serrated adenoma
5	Female	53	Ascending colon	25	1	Yes	No	Sessile serrated adenoma
6		53	Ascending colon	10	1	Yes	No	Sessile serrated adenoma
(*)	Male	60	Cecum	20	1	Yes	No	Sessile serrated adenoma
7	Female	52	Transverse colon	20	2	Yes	No	Sessile serrated adenoma
8	Female	54	Transverse colon	15	1	Yes	No	Sessile serrated adenoma
9	Female	51	Ascending colon	25	2	Not available	No	Sessile serrated adenoma
10	Female	72	Hepatic flexure	25	1	Yes	No	Sessile serrated adenoma
(*)		72	Ascending colon	35	5	Not available	No	Sessile serrated adenoma
11	Male	54	Ascending colon	12	1	Yes	No	Sessile serrated adenoma
12	Female	53	Ascending colon	10	1	Yes	No	Sessile serrated adenoma
13	Female	42	Ascending colon	25	4	Not available	No	Sessile serrated adenoma
14	Male	57	Ascending colon	30	5	Not available	No	Sessile serrated adenoma

(*) indicates the same patient as in the preceding row.
